# Overcoming Challenges in Interdisciplinary Collaboration Between Human and Veterinary Medicine

**DOI:** 10.3390/vetsci11110518

**Published:** 2024-10-23

**Authors:** Louise Han, Yerhee Lee, Hyunsu Lee, Hyejin Lee, Jeong-Ik Lee

**Affiliations:** 1 Regenerative Medicine Laboratory, Center for Stem Cell Research, Department of Biomedical Science and Technology, Institute of Biomedical Science and Technology, Konkuk University, Seoul 05029, Republic of Korea; lhan2025@chadwickschool.org (L.H.); yrilee@ucdavis.edu (Y.L.); lee5747@konkuk.ac.kr (H.L.); 2 Chadwick International School, 45 Art Center-Daero 97 Beon-Gil, Yeonsu-gu, Incheon 22002, Republic of Korea; 3 Animal Biology, College of Agriculture and Environmental Science, UC Davis, 1 Shields Ave., Davis, CA 95616, USA; 4 Department of Veterinary Obstetrics and Theriogenology, College of Veterinary Medicine, Konkuk University, Seoul 05029, Republic of Korea; hyejinly@konkuk.ac.kr

**Keywords:** one health, interdisciplinary collaboration, translational medicine, companion animals

## Abstract

Companion animals, such as dogs and cats, have gained considerable attention in translational medicine due to their potential as models for human diseases. The use of these animals in research has opened new avenues for developing treatments that can benefit both human and veterinary patients, aligning with the One Health approach. Unlike traditional laboratory models like mice, rats, and rabbits, companion animals naturally develop diseases that closely mirror those in humans, including but not limited to diabetes, aging, cancer, and neurological disorders, making them particularly valuable in translational research. Recent advances have highlighted the role of companion animals in enhancing the effectiveness of novel therapies during clinical trials, as they are exposed to diverse environmental and lifestyle factors similar to those experienced by humans. However, the integration of companion animals into translational medicine presents challenges, particularly in terms of collaboration between veterinary and human medicine, where terminology differences in anatomy, clinical terminology, and animal classification can lead to miscommunication. In conclusion, these findings underscore the need for better implementation of the One Health approach by uniting the fragmented collaboration between veterinarians and doctors through interdisciplinary training and fostering unified efforts across both fields, with experts from various disciplines contributing their specialized knowledge in clinical practice and research.

## Simple Summary

This report explores the challenges of translating new therapeutic drugs from preclinical studies to human clinical trials, with a focus on the limitations of using traditional laboratory animals like mice and rats. Despite their widespread use in disease research, these animals often fail to accurately predict human outcomes, leading to a high failure rate in clinical trials. To bridge this gap, we discuss the potential of using companion animals that naturally develop chronic conditions similar to humans, such as dogs and cats, as more relevant models for human diseases. The report also underscores the significance of enhancing collaboration between veterinarians and doctors to address potential communication challenges and fully leverage the potential of companion animals in research. By fostering this interdisciplinary approach, we can improve the success rate of clinical trials and advance healthcare for both humans and animals.

## 1. Introduction

Translating novel therapeutics to enhance patients’ survival and quality of life remains difficult as many investigational drugs do not transition from preclinical to human clinical trials. While mice and rats, which represent approximately 95% of all laboratory animals, have certainly advanced our understanding of disease progression and helped devise treatments for human diseases, they frequently proved to be unreliable in predicting the outcomes of clinical trials [1]. Approximately 89% of novel drugs do not pass human clinical trials, with half of those failures due to unanticipated human toxicity [2]. A notable example was that AN-1792 vaccine trials for Alzheimer’s disease enhanced the production of Aβ antibodies in patients but did not effectively remove Aβ deposits from the brain or slow cognitive decline [3]. During P41~53 phase IIa clinical trials, some patients developed meningoencephalitis, possibly due to antibody and T-cell infiltration of the brain, which was not predicted by experiments on transgenic mice because of their genetic uniformity and differences in plaque composition [4]. As a result, laboratory animals that are extensively inbred, kept in closely regulated conditions, and markedly different from humans may not accurately mirror the complex genetic, environmental, and physiological diversity present in humans, and so the effectiveness of drug treatments [1].

To bridge the gap between preclinical and human clinical trials, incorporating companion animals into translational medicine through the One Health framework offers a more integrative approach that benefits both veterinary and human patients through close collaboration between veterinarians and doctors. There are various reasons why companion animals may be valuable. Firstly, these animals naturally develop diseases that closely resemble human conditions, making them highly relevant models for studying these diseases and testing potential treatments [5]. Secondly, assuming companion animals are kept at a close distance to their owner for mainly entertainment, interest, affection, or for show or sporting events [6], companion animals experience the unique environment and lifestyle of their owners, which reflects the environmental variability in humans. Thirdly, companion animals are not subject to the same degree of as laboratory animals, making their involvement in drug trials a potentially less ethically controversial alternative. Finally, similar to humans, the life expectancy of companion animals has increased due to advancements in veterinary medicine, preventative care, and better nutrition. In many ways, veterinary care parallels human healthcare. Companion animal owners now seek comprehensive veterinary services, including regular health screenings, access to specialized care, and advanced diagnostic tools. For instance, over the past 20 years, the veterinary field became increasingly specialized, with 22 AVMA-recognized veterinary specialty organizations [1]. This mirrors the specialties seen in human medicine.

Considering these advantages, companion animals could facilitate a more seamless transition between preclinical trials involving laboratory models (e.g., mice and rats) and clinical trials, benefiting both human and companion animal patients affected by similar diseases [1]. Achieving synergy requires close collaboration and communication between doctors and veterinarians, given that the two medical disciplines have convergence and the same aim of betterment of the health of their respective subjects, humans or animals. This aligns closely with the One Health approach, which emphasizes the interconnectedness of animal and human health to enhance overall health outcomes. Yet, the process can be hindered by nuances in clinical terminology that could result in potential non-technical errors as well as by disinterest in or time constraints involved in communication between veterinarians and physicians. This commentary discusses specifically how different understandings of directional anatomy, anatomical terms, and animal size classification depending on the field can lead to miscommunications and errors in research and clinical practice. Additionally, this commentary aims to thoroughly assess the role of companion animals as disease models that are shared between human and veterinary medicine and explore the challenges in collaboration and communication between the two fields, which will be discussed through the lens of the One Health approach.

## 2. One Health Strategy and Its Shortcomings

The One Health strategy acknowledges the interdependence of human, animal, plant, and environmental health on both local and global scales [7]. It employs a holistic approach by fostering and enhancing cross-disciplinary collaboration, integrative research, capacity building, clinical practice, policy development, and communication among a wide range of stakeholders, such as doctors, veterinarians, dentists, nurses, and professionals from other health and environmental science fields [8].

The One Health strategy originates from the One Medicine concept by the 19th-century German physician and pathologist Rudolf Virchow, who believed in collaboration among experts in human and veterinary public health to tackle issues related to zoonotic diseases [9]. Virchow coined the term zoonosis and stated that “between animal and human medicine there are no dividing lines–nor there should be” [10]. This philosophy of interconnected health disciplines has historically manifested in collaboration between human and animal medicine in areas like zoonosis and zoopropyhylaxis, antibiotic resistance, and vaccines. One of the best-known early efforts was Edward Jenner’s work with cowpox, which demonstrated the principle of using animal-derived material to confer immunity in humans, establishing the foundation for modern vaccination programs [11].

As zoonotic diseases become more prevalent in the 21st century, many researchers have brought up the need for integration of the One Health strategy, as 60% of infectious agents that infect humans are zoonotic [12]. As a result, multiple world organizations, such as the World Health Organization (WHO), the World Organization for Animal Health (OIE), and the Food and Agriculture Organization (FAO), have called for a closer alignment between human, animal, and environmental health sectors. For example, antibiotic resistance, which is a challenge that stems from the improper and excessive use of antibiotics in veterinary and human medicine, has prompted the antimicrobial (AMR) surveillance process to monitor resistance trends in both human and animal populations [13]. However, despite these renewed efforts, it must be noted that collaboration between veterinarians and doctors has often been sporadic due to lack of inter-sectoral communication and lack of inter-sectoral trust [14]. The vision for One Medicine, which thrived in the 19th century, began to fade in the early 1900s as many veterinary schools shifted their focus towards agriculture [9].

The COVID-19 pandemic revealed the limitations of keeping human and non-human animal health siloed, emphasizing the need for a better implementation of One Health. Notably, veterinarians questioned their omission in the COVID-19 response despite their considerable expertise and knowledge in disease control [15,16]. Veterinarians criticized questionable management strategies used, such as relying on flawed mathematical models, delaying deaths to manage the healthcare system’s capacity, and lacking veterinary practices such as biosecurity and prevention [15,16]. A semi-constructed interview with academic experts who have or had active involvement in One Health revealed that there is a lack of a clear definition and resulting for One Health, and isolated approaches taken by various sectors hinder professionals’ ability to collaborate effectively across disciplines [14]. This disconnection is also evident in the medical industry, where, despite sharing a certain common ground for clinical language and the shared goal to improve patient outcomes, doctors and veterinarians rarely communicate with each other [17]. As a result of these limitations, in high-stake situations where veterinarians and doctors are required to collaborate, such as emerging disease outbreaks or addressing prevalent health issues, the effectiveness of response efforts can be compromised, with more specific information discussed in Section 5.

## 3. Companion Animals and One Health

While zoonotic diseases have long been a focus of One Health, companion animals also play a significant role within this framework. Companion animals often receive a level of care that mirrors human healthcare systems. For example, companion animal owners often pursue comprehensive veterinary care and opt for long-term treatment of chronic conditions [1]. Companion animals, more specifically dogs and cats, spontaneously develop a wide range of diseases that are similar to those seen in humans, including but not limited to diabetes, obesity, cancer, neurological diseases, aging patterns, etc. [18,19,20,21]. In addition, companion animals have shared environmental and socioeconomic risk factors, while having a shorter life span with a more rapid disease progression [22]. This spontaneous development of analogous diseases in companion animals has created an opportunity for cross-disciplinary collaboration in translational medicine in an effort to accelerate novel drug development, fostering reciprocal and shared benefits between animal and human health.

### 3.1. Cancer

Dogs develop several types of cancer that are similar to those arising from humans, including sarcoma, hematological malignancies, bladder cancer, intracranial neoplasms, and melanoma [23]. This makes them a unique model for cancer as many cancers arising in dogs have similar clinical signs, microscopic appearances, and genetic and biological behavior to human cancers [22]. Out of the many types of cancer, mammary tumors are the most frequently diagnosed neoplasm in both female dogs and women and a leading cause of death for both species [24]. Canine mammary tumors (CMTs) and human breast cancer (HBC) are molecularly similar as estrogen receptor status and HER-2 expressions are considered in diagnosis and similar tumor gradings are used [24]. Additionally, factors influencing disease outcomes, such as tumor size, stage, and lymph node involvement, are comparable in HBC and CMTs, making dogs an excellent model for studying the disease and testing new therapies, differentiating themselves from chemically developed models like mice [25].

Likewise, osteosarcoma (OSA), a form of bone cancer in dogs, mirrors the human condition in its aggressive nature and limited treatment options [26]. Clinical and molecular evidence indicate there are similar characteristics, including the tumor’s location, microscopic metastatic disease at the time of diagnosis, the emergence of chemotherapy-resistant metastases, and alterations in the expression or activation of various proteins [27]. Dogs provide a relevant model for enhancing the translational potential of research findings involving cancer, and offer insights into new chemotherapeutic agents and immunotherapies that could benefit both human and canine patients [28].

### 3.2. Diabetes

There has been sufficient evidence for the influence of environmental and genetic factors in canine and feline diabetes mellitus (DM). Diabetes in dogs closely resembles type 1 diabetes in humans, with most of the affected animals showing no detectable levels of insulin and pancreatic analysis typically reveals a total absence of islets [29]. While genetically inbred rodent and large animal models subjected to chemical or surgical induction limit the replicability of results in humans, the incidence of type 1 diabetes occurs between 0.4% and 1.2% and is markedly elevated in specific breeds that are genetically predisposed [30,31]. Both human and canine DM not only show similarities in conventional treatment (life-long exogenous insulin therapy to maintain glucose homeostasis), but also show similarities in pancreatic islet physiology, size, and cellular composition [32].

Meanwhile, diabetes in cats closely resembles type 2 diabetes in humans in clinical, physiological, and pathological aspects. Shared pathological features include the formation of amyloid deposits in the islets, reduction of around 50% in beta cell mass, and complications including peripheral polyneuropathy and retinopathy [33]. Both conditions typically onset in middle age and are linked with obesity. Like human diabetes, high-carbohydrate diets may play a role in predisposing cats to obesity and diabetes due to increased blood glucose and insulin levels [34]. Collaboration with existing human transplant programs and companies with innovative technologies will play a key role in leveraging the dog and cat diabetes model to improve diabetes treatment for both animals and humans. As the incidence of diabetes in pets increases alongside humans, these companion animal models offer a valuable bridge for assessing new therapeutic interventions and the investigation of long-term complications [35,36].

### 3.3. Neurological Diseases

Companion animals showed that they naturally inherit or acquire neurological diseases, conditions like epilepsy, metabolic disorders, brain tumors, spinal cord injuries, and stroke [20]. One of the most pervasive neurological conditions affecting both humans and dogs is epilepsy, with a similar prevalence and causes, including brain tumors, encephalitis, and neurodegenerative diseases [20]. Notably, canine epilepsy shows similar seizure phenotypes and electroencephalography (EEG) patterns to humans, with comparable cognitive and behavioral comorbidities such as anxiety and cognitive alterations [37]. Furthermore, historically, seizures induced in laboratory dogs have been a crucial resource in progressing new treatments for epilepsy, as the development of vagus nerve stimulation (VNS) for drug-resistant epilepsy in humans was based on research involving dogs with induced seizures [38]. Considering that resistance to antiepileptic drugs continues to be a major obstacle in treating epilepsy in both dogs and humans, dogs may provide valuable insights when bridging the gap between preclinical and human clinical trials, being valuable for testing novel anticonvulsant drugs and devices.

Additionally, with the increase in the aging population worldwide, age-risk-associated disorders like Alzheimer’s disease have become a rapid public health concern. Similarly, canine cognitive decline (CCD), which exhibits numerous parallels with Alzheimer’s disease in humans, is frequently seen in senior dogs and is a leading factor in morbidity among companion animals [39]. Similar to humans, older dogs show signs of oxidative damage, caspase activation, astrogliosis, cortical shrinkage, and reduced brain volume [40]. As a result, neuropathological evidence supports utilizing older dogs as a model for studying human aging and dementia. Research shows dogs may possess greater predictive accuracy in evaluating cognitive-enhancing therapeutics compared to chemically induced mice models, given the dogs’ ability to accurately reproduce the outcomes observed in human clinical studies with first-generation ampakine and CP-118,954 [40].

### 3.4. Aging

The companion dog offers a unique opportunity to combine two key approaches in aging research: one, uncovering the genetic and environmental factors influencing aging and age-related diseases, and two, discovering the underlying mechanism within a practical time frame [41]. Regarding the first approach, companion dogs share similar environments with humans, exposing them to the same pollutants, diets, and lifestyles, which makes them relevant models for environmental impacts on aging [21]. Regarding the second approach, an analysis of morbidity and mortality data from 112,375 humans in the U.S. Census Bureau’s National Longitudinal Mortality Study, 37,480 dogs in the Veterinary Medical Data Analysis, and 5095 dogs in the VetCompass Programme revealed that both humans and dogs experience near identical age trajectories in cancer-related deaths and similar age-specific patterns in causes of death related to congenital and metabolic conditions, with metabolic conditions in both species often linked to lifestyle factors like obesity [21]. Additionally, a clinical trial with 24 middle-aged healthy dogs revealed that rapamycin, a drug approved by the FDA for clinical use in preventing organ transplant rejection in humans, showed improvements in both diastolic and systolic heart function related to aging, highlighting the potential of canine models for studying and testing drugs that may potentially delay aging and improve health span [42].

### 3.5. Others

Although not discussed in this article, companion animals have further implications in the fields of regenerative medicine, chronic obstructive pulmonary disease, chronic pain, and osteoarthritis [43,44,45,46]. Additionally, while our definition of companion animals primarily focuses on dogs and cats, it is important to acknowledge that other animals such as miniature pigs and horses may also be denoted as companion models in research. For example, horses naturally develop asthma, which has strong similarities to human asthma, making them valuable for studying this condition [47]. Minipigs, on the other hand, have been utilized in research on various conditions that resemble human conditions, including metabolic conditions like dyslipidemia and atherosclerosis, and skin injuries and tissue damage caused by radiation exposure [48].

In addition, it is important to note that large animal models that are not considered as companion animals can also play a crucial role when bridging the gap between preclinical and human clinical trials. While companion animals offer valuable insights due to their genetic similarities and shared environments with humans, large animals can be better suited for modeling human exposure to certain environmental conditions and physiological stressors.

Particularly, livestock may be important in replicating specific agricultural environments that mirror human occupational settings. In intensive farming conditions, pigs are frequently exposed to poor air quality with high levels of ammonia, dust, and microbial pollutants, especially during winter seasons when ventilation rates are low. The importance of ventilation in intensive swine farms was revealed as high concentrations of airborne pollutants can weaken piglet immunity and cause respiratory diseases [49]. These conditions are comparable to respiratory issues seen in swine barn workers, who often suffer from higher incidences of lung dysfunction due to airborne pollutants [50]. The specific environmental challenges faced by livestock in these farming setups provide insights into occupational health risks for humans that companion animals, typically living in household environments, would not experience.

Another example is radiation exposure, where livestock such as cattle provide insights into the effects of toxins introduced to the environment. After the Fukushima nuclear disaster, a study revealed that cattle exposed to radioactive materials such as iodine-131 at farms near the Fukushima Daiichi nuclear plant showed slight thyroid hyperactivity, elevated hormone levels, and cortisol levels [51]. These effects closely mirrored those seen in humans, where children below 15 years of age exposed to radiation from the Chernobyl event showed a heightened risk of thyroid cancer [51]. The cattle’s continuous exposure to radioactive materials through the environment made them better suited for studying the long-term impacts of environmental stressors.

Therefore, veterinary clinical trials should not be constrained to companion animal models alone; the selection of the most appropriate model should be carefully assessed based on the specific research context, as certain models can offer deeper insights into environmental factors that may not be fully replicated in companion animal studies.

### 3.6. Limitations to Companion Animal Model

While companion animals serve as valuable models in translational medicine, the alignment between human and animal disease models, especially in cancer research, can vary significantly [25]. Some cancers in companion animals may not fully mirror human conditions, such as different incidences of cancer types in humans and animals and species-specific differences [22].

For instance, Feline Oral Squamous Cell Carcinoma (FOSCC), while sharing histopathological similarities with human head and neck squamous cell carcinomas (HNSCCs), exhibits a higher rate of local invasion and recurrence, making it less useful for testing human therapies [22]. Similarly, canine oral melanoma (COM) provides a model for melanoma research, but differences in immune responses and metastatic patterns present challenges for translational study [22].

The companion animal model is still a relatively new concept. Rather than suggesting that companion animals lead to translational failures in human medicine, it is more accurate to acknowledge that there are inherent limitations when using these animals as models for certain diseases. Biological, physiological, and clinical behaviors can vary significantly between species, and researchers must account for these differences when selecting an appropriate model, as certain treatments or disease progressions may not be fully mirrored in humans. Furthermore, while some studies suggest that specific companion animal models may better represent particular diseases—such as diabetes in dogs reflecting type 1 diabetes and in cats reflecting type 2—it is important to recognize that our understanding of both human and animal diseases is still evolving. As a result, it remains challenging to definitively align certain species with specific diseases.

## 4. Ethical Concerns of Involving Companion Animals in Translational Medicine

Ethical concerns in veterinary clinical trials involving companion animals must be addressed with particular sensitivity to the bond between these animals and their owners. Unlike preclinical studies where diseases are artificially induced and animals may be sacrificed, veterinary clinical trials should focus on studying naturally occurring conditions in animals where owners are seeking veterinary care [1].

The ethical framework for such trials must involve rigorous oversight to protect the interests of both the animals and their owners. Since animals cannot provide consent, their owners must make informed decisions on their behalf. This process should be guided by ethical standards, with each study being examined by the Institutional Animal Care and Use Committee (IACUC), so that all ethical considerations are thoroughly evaluated. The IACUC serves an essential function in safeguarding the welfare of the animals by ensuring that all procedures are justified and humane [52]. An informed consent process similar to that of human patients should also be employed to avoid conflicts of interest, ensuring that all standard care options are discussed before a trial is considered [1]. During this process, veterinarians and researchers must be transparent about any potential risks to the health of animals, highlighting the trial-like nature of research with uncertain outcomes because there are many uncertainties about whether a companion animal will be helped or harmed.

Based on the guidelines set by the IACUC, the standards for laboratory animals and companion animals in clinical trials highly contrast and should not be treated similarly. Laboratory animal research involves animals that are not privately owned, which places the responsibility for ethical standards solely on the research institution and oversight bodies like the IACUC. These animals are protected by stringent regulations, such as the Public Health Service Policy and the Animal Welfare Act, which enforce oversight through ethical reviews and harm–benefit analyses to ensure the animals’ welfare [52]. In contrast, veterinary clinical trials involve companion animals that are owned by a human being, meaning the process differs. As mentioned above, owners must provide informed consent for their pets to participate in studies, and their understanding of the risks and benefits is essential. The American Veterinary Medical Association’s “Establishment and Use of Veterinary Clinical Studies Committees” (VCSC) policy emphasizes the need for the VCSC to oversee the informed consent process, assess compensation for owners, and evaluate study-related risks and benefits to the animals [53]. Additionally, it is indicated that the VCSC should consist primarily of veterinarians who are actively engaged in clinical practice and collaborate closely with the IACUC [53]. It is evident that the ownership factor significantly changes the ethical landscape for companion animals compared to laboratory animals.

Regarding potential ethical issues in this emerging field, the current popular reference of companion animals in research as “models” may result in public criticism, especially considering that the use of companion animals in research is already a controversial issue [54]. When these animals are involved in research, the public is often concerned about their well-being and the ethical implications of their use. Labeling these animals as “models” could potentially intensify these concerns, as the name itself suggests that they are being held at a level similar to animals used in preclinical research, where the ethical standards are often perceived as less stringent and reduced to benefit solely humans. However, it should be clarified that the label may not be as problematic as it appears because trials involving companion animals will only be pursued when there is potential for companion animals to benefit directly. Because companion animals are co-beneficiaries, it distances them from the traditional “model” paradigm. In this regard, we hope to clarify that our use of the term “model” is strictly a technical reference. This means that while companion animals may present similar illness characteristics and treatment outcomes to humans, it does not imply that they can be used without consideration for their own health and well-being. However, given this potential concern for confusion, it seems necessary to reserve a different language in the future for these companion animals employed in veterinary clinical trials that reflects the ethical commitment to their welfare.

## 5. Potential Misconceptions Between Human and Veterinary Medicine

With the growing interest in companion animal models in translational medicine, effective collaboration between veterinary and human medicine is anticipated to be crucial in advancing healthcare for both humans and animals. However, potential misconceptions between these two fields may hinder seamless communication and collaboration. Specifically, misunderstandings can arise from differences in anatomical terms, directional anatomy, distinctions in the classification of animal sizes, and distinction between clinical signs and symptoms terminology.

### 5.1. Differences in Directional Anatomy Terminology

The terminology used to describe anatomical directions in humans and non-human animals often leads to misconception due to their bodies’ different orientations. While some anatomical terms overlap between human and veterinary patients, humans, being bipeds, stand vertically with their heads oriented at a right angle to their bodies, while quadrupeds have a horizontal body orientation, with their heads aligned in the same direction as their bodies, leading to terminological differences.

Most notably, in human anatomy, the frontal plane, also referred to as the dorsal plane among veterinarians, runs vertically from the head to the feet and divides the body into “anterior”, which refers to the front of the body or the side with our face and belly, and “posterior”, which refers to the back of our body or the side with the back of our head and our behind [55]. In contrast, for quadrupeds like dogs, the frontal plane still divides the body into front and back sections but does so in a way that corresponds with the animal’s horizontal orientation. Because of the animal’s posture, with the spine oriented horizontally, the use of “dorsal” and “ventral” becomes more significant. The dorsal side refers to the upper side of the animal along the backbone, and the ventral side refers to the lower side along the belly [55].

**Figure 1:**
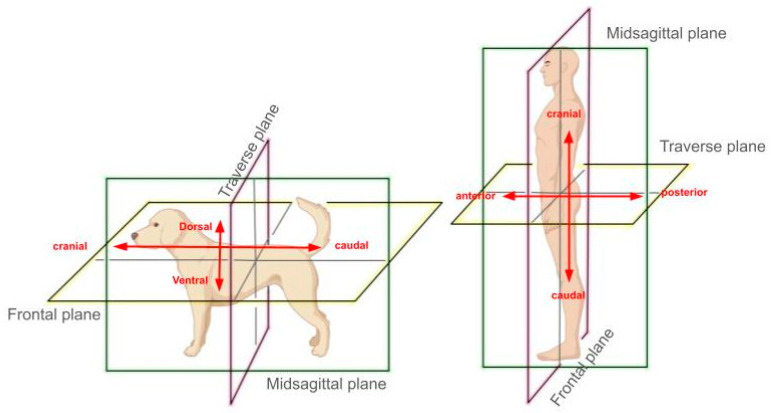
Directional anatomy in bipeds versus quadrupeds.

To refer to the “anterior” and “posterior” sides of quadrupeds, “cranial” and “caudal” are used, which is visually illustrated in Figure 1. “Cranial” points towards the head, but along the animal’s horizontal axis, making it synonymous with the forward, or anterior, side of a human. Conversely, “caudal” refers to the direction towards the tail, aligning with the posterior, or back, of a human.

This difference becomes apparent between veterinary and human medical practitioners. In humans, a frequent knee injury is called an ACL (anterior cruciate ligament) tear, whereas in dogs, the equivalent injury is called a CCL (cranial cruciate ligament) tear because the term “anterior” is not appropriate for quadrupedal species [56]. These distinctions are crucial to accurately describe the location of injuries, diseases, or surgical sites in relation to the body, highlighting the need for specialized expertise in both veterinary and human medicine to avoid errors in treatment and communication.

### 5.2. Defining Small Animals Versus Large Animals

In veterinary medicine, the distinction between small animal and large animal practice is fundamental to how veterinarians approach the treatment and care of different species. Small animal veterinarians typically focus on companion animals, such as dogs, cats, and small mammals like rabbits and guinea pigs. These animals are often categorized as “small animals”. Meanwhile, large animal veterinarians primarily work with livestock and equines, such as cattle, horses, sheep, goats, and pigs, which are classified as “large animals”. However, this classification system does not apply to translational research and animal models, as large animals are referred to as species larger than common laboratory rodents, such as mice and rats. For example, dogs are referenced as “large animals” in this context, not because of their size relative to livestock, but because they offer a more relevant physiological model and better stimulation for human responses [57,58,59,60].

This divergence in terminology can lead to errors in non-technical skills that have far-reaching consequences in research and clinical practice. Non-technical skills (NTSs) refer to a collection of general cognitive and interpersonal abilities, demonstrated by both individuals and teams, that complement technical skills during the execution of complex tasks [61]. Effective communication is acknowledged as a fundamental non-technical skill, encompassing methods to share information. Communication failure is the primary cause of accidental patient harm; a recent analysis examining 2587 sentinel medical adverse events, examined by the US Joint Commission over three years, found that communication was a contributing factor in more than 68% of the cases [62]. With communication being rare between doctors and veterinarians [17], in situations where both professions need to collaborate in a clinical field, these communication errors may be further exacerbated.

For instance, when veterinary studies refer to findings in “small animals”, human medical researchers may interpret it as referring to rodents, the default “small animals” in their field. In emergency situations such as outbreaks of foot-and-mouth disease (FMD) or avian influenza (AI), experts from various disciplines will be deployed to the front lines of biosecurity measures [63]. These scenarios require immediate and coordinated quarantine such as culling infected animals or administering vaccines [64]. In such high-stress environments, non-technical errors stemming from small misunderstandings can lead to significant incidents.

Such issues are problematic in the context of translational medicine or zoonotic research, where the precise transfer of knowledge from animal models to human applications is essential. Aligning understanding across disciplines is crucial for the effectiveness of the One Health strategy, emphasizing the need to strengthen the connection between human and veterinary medicine.

### 5.3. Anatomical Differences Between the Knee and Stifle

It is known that the fundamental structure and arrangement of diarthrodial joints are similar in all mammalian species and share the same purpose of mitigating substantial mechanical forces produced by locomotion [65]. In human anatomy, the knee is a synovial hinge joint composed of the tibiofemoral and patellofemoral parts, supported by structures like the menisci, cruciate ligaments, and collateral ligaments [66]. It links the distal femur with the proximal tibia, facilitating movement and stability [67]. In quadruped mammals, the equivalent structure is known as the stifle joint in their hind legs rather than the joints located in their forelegs, which were assumed to be the knee due to its anatomical position and visual resemblance [68]. Specifically, the cranial and caudal cruciate ligaments are equivalent to the human anterior (ACL) and posterior cruciate ligament (MCL).

Similarly, the carpal bones in dogs are often mistakenly assumed to be analogous to the human knee due to their role in weight-bearing and the anatomical location of the forelimb. However, the quadruped carpus consists of several small bones and ligaments that contribute to the flexibility and movement of the forelimb, much like the wrist in humans. For example, the canine carpus, composed of bones arranged in a proximal and distal row, shares a similar configuration with the human wrist, which allows for a range of motions, including flexion, extension, and slight rotation, which are crucial for locomotor activities [69,70]. These distinctions underscore the importance of precise anatomical understanding to avoid misconceptions that could impact diagnosis and treatment in translational medicine.

### 5.4. Distinction Between Clinical Signs and Symptoms

Another important distinction between the two medical fields is the use of clinical signs and symptoms. Clinical signs are observed by healthcare providers during a physical examination or through diagnostic testing, while symptoms are the subjective experience reported by the patient. Symptoms are only present in human medicine as the patient can feel and communicate to healthcare providers feelings such as pain, nausea, and dizziness. These are instrumental observations that the patient can directly report on. Therefore, they are essential when it comes to making a diagnosis. While animal patients can show symptoms through signs of pain, they cannot self-report their symptoms verbally, meaning veterinarians solely rely on clinical signs to make accurate diagnoses and treatment plans.

It is essential that doctors and veterinarians collaborating in translational medicine clearly distinguish between these terms to avoid potential non-technical errors. The clear distinction in terminology evidences this as follows: vomiting is a symptom in human medicine and a clinical sign in veterinary medicine. Misusing these terms to refer to different species can lead to misunderstandings that hinder collaboration. Accurate terminology ensures effective communication, reducing errors and enhancing diagnosis and treatment when human doctors and veterinarians work together.

## 6. Conclusions: Moving Forward

The growing recognition that many health challenges and chronic diseases are shared between humans and animals, especially companion animals, presents a significant opportunity for interdisciplinary collaboration between veterinary and human medicine. However, the One Health initiative has highlighted some important shortcomings. A significant barrier to achieving this collaboration is the fact that the partnership between veterinarians and doctors remains largely incidental and fragmented, leading to complications where terminological differences may lead to non-technical errors in communication between both disciplines.

To effectively bridge this gap, cross-disciplinary training in One Health concepts is crucial for medical and veterinary students to foster collaboration and a mutual understanding of terminological differences from the earliest stages of professional education. This benefits both professions in the long run, as experts with diverse backgrounds, such as doctor–veterinarians, will likely become key stakeholders in addressing complex and interdisciplinary issues in translational research and in responding to emergent situations such as zoonotic disease outbreaks.

Firstly, incorporating One Health-related courses into the core curriculum that cover shared topics such as zoonotic diseases and comparative medicine will allow students to explore the interrelationship of human, animal, and environmental health. Incorporating case studies, joint projects, and clinical simulations into the curriculum would enable students to work collaboratively, learning to understand the different terminologies and anatomical perspectives outside of their disciplinary community.

Secondly, joint workshops, symposiums, seminars, and conferences can provide platforms for students and professionals to engage in dialogue, share knowledge, and address common challenges. This approach not only enhances mutual respect and communication between future doctors and veterinarians but also prepares them to work effectively in interdisciplinary teams, ultimately improving patient outcomes across both human and veterinary medicine.

Thirdly, a more sustained collaboration is required to prevent non-technical errors caused by the terminological differences between the fields, such as establishing interdisciplinary academic associations focused on sharing terminology and addressing translational medicine challenges. One of the key functions could include a standardized terminology committee that can work towards developing a shared language when working with veterinarians and doctors in translational medicine. This can help promote the exchange of knowledge and practices between the two fields. It is recommended that government-led initiatives, with relevant departments, take the lead. Furthermore, international collaboration will further enhance the exchange across borders. In the end, it is important to note that translational medicine is a broad and evolving field, meaning these are just potential suggestions; it requires adaptable approaches rather than rigid frameworks.

Finally, establishing specific research funding for projects that involve both veterinary and medical specialists would further encourage collaboration and advancements in areas such as pharmacokinetics, toxicology, and differential therapies between small and large animals.

In this increasingly interconnected world, veterinarians and doctors have a profound responsibility to safeguard the health of both people and animals, reinforcing the importance of unified, cross-disciplinary efforts in all areas of health.

## Data Availability

No new data were created or analyzed in this study. Data sharing is not applicable to this article.
